# P-1581. Low Barrier Advocacy: A Weekly Call Script Program to Promote Physician Engagement in Infectious Disease Policy

**DOI:** 10.1093/ofid/ofaf695.1760

**Published:** 2026-01-11

**Authors:** Kelly Dyer, Corey Watts, Kevin Kohm, Elle Saine

**Affiliations:** University of Pennsylvania Perelman School of Medicine, Philadelphia, Pennsylvania; University of Pennsylvania, Philadelphia, PA; University of Pennsylvania, Philadelphia, PA; University of Pennsylvania, Philadelphia, PA

## Abstract

**Background:**

Physician voices are essential in shaping policies that support public health, evidence-based medicine and equitable access to care. Previous advocacy efforts by IDSA have resulted in meaningful advocacy wins in the field of infectious diseases (ID) such as the G0545 billing code, highlighting the importance of physician advocacy engagement. Many ID physicians want to engage in advocacy but face barriers, such as time constraints and uncertainty about how to begin.

**Graph 1.**
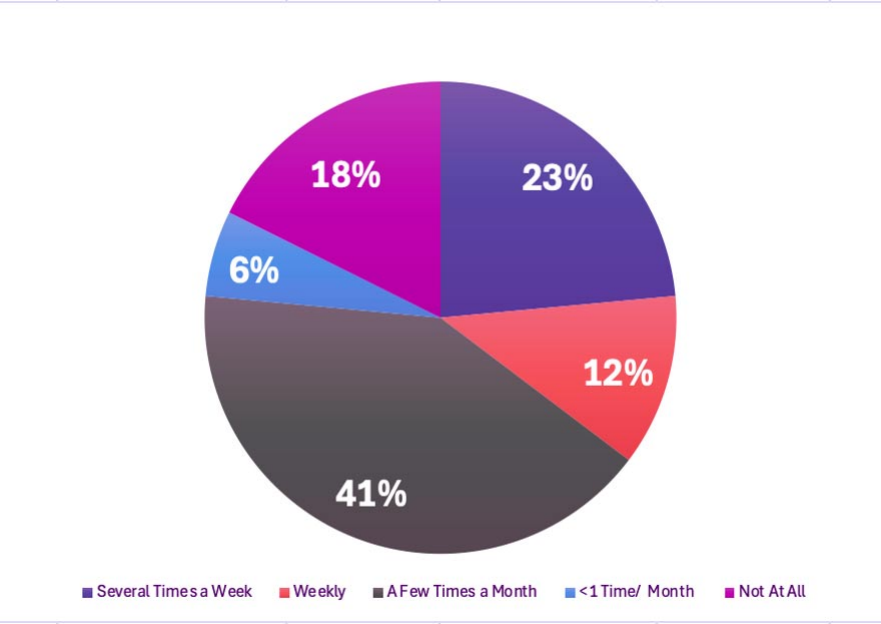
Respondent Call Frequency To Legislators Following Implementation of Call Script Program

**Graph 2.**
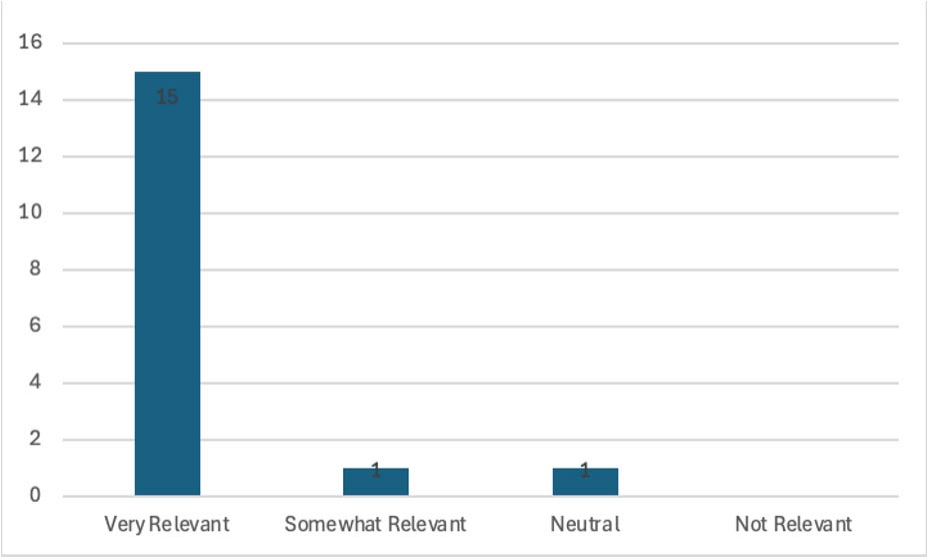
Perceived Relevance of Call Script Topics by Call Script Users

**Methods:**

In January of 2025, we developed a low-barrier advocacy initiative with the goal of increasing ID provider engagement with legislators on topics relevant to patients and providers. Each week we wrote and distributed a concise call script about a topic affecting ID clinical care, research and/or public health. Each script included context about the topic, bulleted options for messaging, and instructions for contacting legislators. Paper call scripts were distributed weekly to ID providers rotating on inpatient consult services in a shared office space. The standing distribution time was chosen to limit disruption to patient care. A QR code on the printed script linked to a digital file with prior call scripts and a form where providers could propose future call script topics. Participation was optional. During week 10 of the program, a 10-question survey was subsequently disseminated to the ID division to assess the reach and impact of the program. Providers were included if they reported receiving at least one script.

**Table 1.**
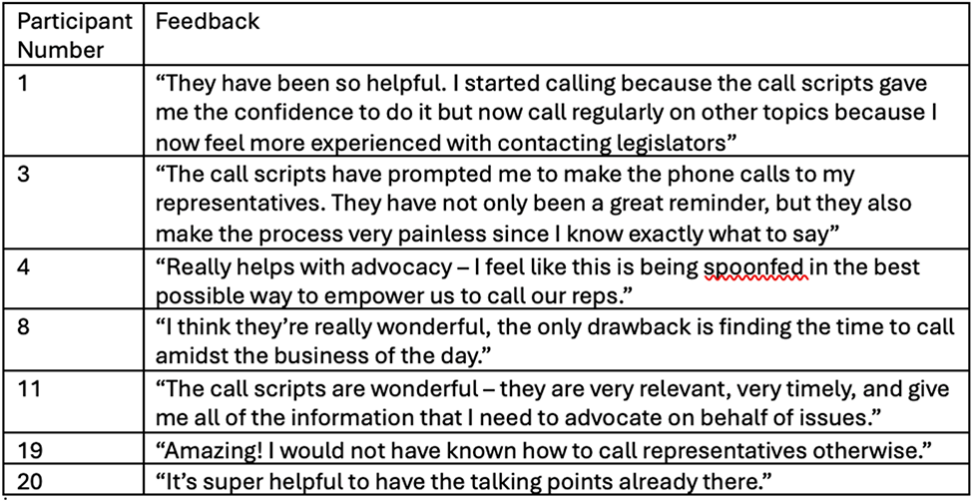
Representative Feedback about the Call Script Program by Call Script Users

**Results:**

To date, the program has distributed 10 call scripts to ID providers across our division. Seventeen ID providers who reported receiving at least one script responded to the survey. Eight (47.1%) reported never having contacted a congressional representative by phone prior to the call scripts. Thirteen (76.5%) reported using the call scripts to contact representatives and 14 (82.4%) reported that the call script made it much or somewhat easier to engage in advocacy. Thirteen (76.5%) reported calling at least monthly since the start of the program.

**Conclusion:**

This low-barrier advocacy program led to increased ID provider advocacy engagement. Weekly call scripts can help mobilize the ID workforce around shared priorities and can help amplify physician voices in policy discussions.

**Disclosures:**

All Authors: No reported disclosures

